# Clinically elevated depression scores do not produce negative attentional biases in caregivers of autistic children

**DOI:** 10.3389/fpsyt.2023.1192669

**Published:** 2023-09-07

**Authors:** Brian Lovell, Kris McCarty, Phoebe Penfold, Mark A. Wetherell

**Affiliations:** Department of Psychology, Northumbria University, Newcastle upon Tyne, United Kingdom

**Keywords:** autism, caregivers, depression, negative attentional bias, dot probe

## Abstract

**Objective:**

Depression scores in caregivers of autistic children often fall in the clinical range. The attention of clinically depressed individuals tends to be biased toward negatively toned information. Whether caring for an autistic child might also be characterized by a negative attentional bias was explored here.

**Methods:**

A sample of *N* = 98 (57 caregivers and 41 controls) completed questionnaires assessing depressive symptoms. Orienting attention to (i.e., vigilance), and shifting attention away from (i.e., disengagement), negative information was assessed via an online version of the emotional face dot probe task.

**Results:**

Mean depression scores in caregivers, falling in the borderline clinical range, were significantly higher compared with controls. Groups, however, were indistinguishable with respect to vigilance and disengagement, and these attentional indices were unrelated to depression scores.

**Conclusion:**

Caring for an autistic child, while associated with borderline clinical depression scores, was not characterized by a negative attentional bias. Findings are discussed in the context of methodological shortcomings and recommendations for future research.

## Introduction

Psychological distress, of which depression is one common marker, tends to be elevated in the context of caring for an autistic child. Indeed, a plethora of cross-sectional research has linked caring for an autistic child with increased depression scores (1–4). The negative psychological impact of caring for an autistic child appears to be enduring. Indeed, caregivers’ use of emotional coping behaviors increases over time, and emotional coping behaviors are established risk factors for depression in this sample (5, 6). Caregivers have also commented in qualitative studies that concern about who will care for the autistic child in the future, at a time when they might be physically unable, cause their feelings of depression to increase over time (7). Caregivers’ depression scores often satisfy the criteria for a clinical case (8, 9). It was recently estimated that clinically significant psychopathology, in the form of depression, is evident in approximately 33% of caregivers of autistic children (10).

Cognitive models of depression implicate difficulties with cognitive control in the etiology and maintenance of the disorder (11). The cognitive schemas of depressed individuals are typically defined by themes of loss, rejection, and failure, and depressed individuals’ attention, guided by these schemas, can become biased toward the processing of negative information (12). In other words, depressed individuals appear to be hypersensitive to negatively toned information in their environment. For example, the emotional face dot probe task presents two faces, one with a negative (i.e., sad) and one with a neutral expression, side by side on the screen and asks participants to indicate, when cued, which face has been replaced by a large red dot. Response latencies, captured in milliseconds, provide attentional bias indices of vigilance (i.e., orienting attention to negative stimuli) and disengagement (i.e., shifting attention from negative stimuli). Several studies have reported that depressed individuals found it more challenging, after receiving a visual cue, to disengage their attention from negatively toned stimuli. In other words, depressed individuals processed negative stimuli for longer periods following fixation. Some evidence, though less prevalent, shows that depressed individuals are also more vigilant to negative stimuli, detecting them faster following visual cues (13–18). This attentional bias toward the processing of negative content, characterized predominately by problems with disengagement, has been replicated with several other exogenous cueing tasks (19). It was the recent conclusion of several researchers, following comprehensive meta-analysis and systematic review, that increased depressive symptomology provides one robust psychological marker for negative attentional bias (20, 21). Researchers have also found that depressed individuals sometimes lack the positive attentional bias evident in non-clinical samples. Indeed, vigilance for positive stimuli (i.e., faces conveying positive expressions such as happiness) was slower, and disengagement much faster, in depressed individuals compared with controls (22–24). Studies using more sophisticated eye-tracking designs, allowing for continuous monitoring of visual attention, have also found evidence for negative attentional bias in the context of depression. Indeed, when presented with facial expressions varying in emotion (i.e., some positive, some neutral, some negative), depressed individuals glanced more often at the negative, and less often at the positive, stimuli compared with controls (25). Total fixation time on negative stimuli, assessed with eye tracking, has also been shown to be longer in depressed individuals (26). The evidence suggests the attention of depressed individuals, and those with conditions characterized by increased depression scores, is biased in favor of negative information. Depressed individuals seem to pick up on negative content faster and, finding disengaging from it more challenging, process it for longer. The reverse appears to be the case for positive content, with depressed individuals identifying it slower and, disengaging faster, processing it for shorter periods.

Depression scores tend to be high in the context of caring for an autistic child, often falling in the clinical range. Whether caring for an autistic child is characterized by a negative attentional bias, however, has not yet been explored. This study aims to fill this gap. Several lines of evidence converge to suggest this might indeed be the case. For example, caregivers of autistic children self-report more attentional errors compared with their non-caregiving counterparts (27). In the lab, using objective, performance-based measures of attentional control, caregivers were found to make more errors than their non-caregiving counterparts (28–30). Much of the research involving objective tests of attention, however, has involved spousal dementia caregivers, and generalizing these findings to caregivers of autistic children, therefore, should be done cautiously. That said, it was the conclusion of researchers following a comprehensive review that caring for individuals with neurological conditions such as dementia is comparable, in terms of psychological demand and burden, to caring for individuals with developmental conditions such as autism (31). Whether the attention of informal caregivers is biased toward the processing of negatively toned information has rarely been explored. Only four studies have been completed to date as far as we can find, with findings showing caring for a relative experiencing chronic pain to be associated with slower disengaging attention from negative (i.e., faces expressing pain), and faster disengaging attention from positive (i.e., faces expressing happiness), stimuli (32, 33). Most recently, researchers found that spousal dementia caregivers were faster, when their anxiety levels were high, at shifting their attention to faces displaying negative (i.e., sad) expressions compared with controls (34, 35). Familial caregiving has also been linked with increased rumination (i.e., excessive thinking about negative thoughts and feelings), and some researchers have implicated ruminative thinking as one psychological marker for negative attentional bias (36, 37). Ruminative thinking and negative attentional bias, each characterized by the prolonged processing of negative information, are closely related constructs (38).

The negative emotional impact of caring for an autistic child has been well-documented, with caregivers’ depression scores often satisfying clinical standards (10). Clinically depressed individuals preferentially attend to negative information, detecting it faster and processing it for longer (13, 18). Attention, as assessed via self-report and objective lab-based measures, tend to be poorer in familial caregivers (27, 29). Negative attentional biases have been detected in caregiving samples that are comparable, in terms of psychological demand and burden, with parents of autistic children (32–34). Whether caring for an autistic child might be associated with an attentional bias for negative information, and whether this bias takes the form of vigilance (faster orienting to) or disengagement (slower orienting away from), will be explored here. This study will be the first, we believe, to assess attentional control biases in the context of caring for an autistic child. It was hypothesized that caregivers of autistic children, scoring higher than controls in depression symptoms, would be faster to orient their attention to, and slower disengaging their attention from, negatively toned stimuli on an online version of the emotional face dot probe task.

## Materials and methods

### Participants

A’priori power analysis configured for medium effect (*f* = 0.25), alpha of 0.08, and with two groups indicated a sample of *N =* 128 would be needed. A sample of *N* = 234 participants, of which 156 were caregivers of autistic children and 77 were controls (i.e., parents of non-autistic children), were recruited via adverts posted in parenting support/information groups on social media. A link to the study survey, hosted by Qualtrics, was included in the recruitment advert. Participation was open to those (a) aged 18 years or older, (b) parenting at least one child aged 3–21 years and living at home full time, and (c) not currently experiencing, or in the last 12 months experienced, any long term stressors (e.g., bereavement, divorce). The child’s autism diagnosis was confirmed by parent report.

Of the *N* = 234 participants that consented to take part, five failed to complete the depression questionnaire and 14 failed to provide code words for matching questionnaire and dot-probe data. These participants were removed, as were two participants with poor quality data (i.e., <30% accuracy) on the dot-probe task (39). Data for another 115 participants who failed to start the dot probe or complete it to the point where data could be used (i.e., <25% missing trials) were also removed (39). Failure to complete the dot-probe task was equally likely for caregivers and controls (*χ*^2^ = 0.74, df = 1, *p* = 0.39). The high attrition rate (58.1%) was not unexpected in light of changes imposed on the study protocol by COVID-19. Our lab space was indefinitely closed due to COVID-19 which meant the dot-probe task, originally intended to be lab-based, needed to be modified to run online. Research has shown retention for online studies, especially those with complex cognitive tasks, are much poorer compared with lab-based studies (40, 41). The final sample taken forward for analysis, therefore, was *N* = 98, and this included 57 caregivers and 41 controls. Sample characteristics by group are presented in Table 1.

**Table 1:** Sample characteristics by group.

	Caregivers (*N* = 57)	Controls (*N* = 41)	*p* =
**Gender**			0.19
Male	8 (14%)	10 (24%)	
Female	49 (86%)	31 (76%)	
**Relationship status**			0.61
Partnered	46 (82%)	32 (78%)	
Not partnered	10 (18%)	9 (22%)	
**Employment**			0.09
Employed	42 (75%)	24 (59%)	
Not employed	14 (25%)	17 (41%)	
**Smoker**			0.61
Yes	6	2	
No	50	39	
Number of children	2.3 ± 0.80	1.9 ± 0.84	0.04
Mean age (years)	41.2 ± 8.2	43.4 ± 8.6	0.21
Mean alcohol (drinks per week)	4.6 ± 7.7	3.1 ± 4.2	0.27
Mean exercise (times per week)	4.0 ± 3.7	4.1 ± 3.5	0.90
Mean sleep (hours per night)	6.4 ± 1.2	6.7 ± 1.1	0.24

### Measures

#### Control measures

Data with respect to socio-demographic (e.g., age, gender, annual income, employment status) and lifestyle (e.g., relationship status, number of children, sleep, exercise, alcohol) variables, known to be influential for study outcomes of interest, were collected. Data with respect to characteristics of the autistic child (i.e., current age, diagnosed age) were also collected to safeguard against spurious relationships emerging.

#### Depressive symptomology

The Hospital Anxiety and Depression Scale (HADS) was used to assess depressive symptoms (42). The HADS is composed of 14 items, seven assessing depression (e.g., *“I feel as if I am slowed down”*) and seven assessing anxiety (e.g., *“I feel tense or wound up”*), with each item scored using a four-point Likert type scale (0, *never* - 3, *always*). Subscale scores for depression are calculated by summing across all seven items, with total scores ranging between 0 and 21. Higher scores are indicative of greater depressive symptoms. Subscale scores falling in the range of 0–7 are deemed normal. Scores ranging from 8 to 10 are indicative of a borderline clinical, and scores >11 are a clinical, case. Internal consistency for the HADS depression subscale was good (*α* = 0.86) in recent studies (43), as was the case here (*α* = 0.85).

#### Emotional face dot probe task

A series of 20 face photographs of 10 different adult models (50% female), 10 of them expressing negative emotions (i.e., sadness) and 10 with neutral expressions, were taken from the Karolina Emotional Recognition Facial Database with the consent of authors (44). Hair and neckline, potentially conveying emotional information, were cropped from each photograph, as was any peripheral information. A series of 10 face pairs standardized for shape (i.e., oval), size (500 × 700 pixels), and background (i.e., black) was the result. The emotional face version of the dot-probe task, using expressive faces as opposed to emotive words, was preferred here, and this was because expressive faces, research has found, convey more affective information than words (23). Faces, therefore, might be particularly sensitive for detecting negative attentional biases. Faces might also be particularly suitable stimuli for parents who are accustomed, until language develops, to decoding the emotional needs of their children via facial cues (45). It has been estimated in fact that 25–35% of autistic children are minimally or non-verbal, and caregivers have reported that facial cues are critical for understanding the feelings and intentions of their children (46).

### Procedure

Clicking the link in the recruitment advert directed participants to the survey collection platform, Qualtrics. Questionnaires assessing the socio-demographic and lifestyle factors, and characteristics of the autistic child were completed first. This was followed by the relevant HADS subscale to quantify depressive symptoms. Participants were then redirected, at survey completion, to an online version of the emotional dot probe task. Instructions for task completion were presented on screen. Participants were advised that two faces of the same person, one displaying negative (i.e., sad) and one with a neutral expression, would appear on the screen in left and right positions for 500 ms, with one face then replaced by a large red dot (i.e., dot probe). Participants were asked to indicate, as quickly as possible, which face had been replaced by the probe, pressing the Z key for the face on the left and the M key for the face on the right. The detection latency for the probe was captured in milliseconds. Accuracy feedback was provided following each trial, with “correct,” for correctly identified probe locations, or “incorrect,” for incorrectly identified probe locations, displayed on screen for 500 ms. The time delay between trials was 1,000 ms. Negative-neutral face pairs accounted for half of the experimental trials and neutral-neutral face pairs the other half, with probes replacing either face with equal probability. Face pairs of the same model, of which there was 10 total, were repeated in presentation 9–10 times for a total of 96 experimental trials. The relative position of each face pair (left or right) was counterbalanced. Face pairs were presented in random order to safeguard against order effects were and always presented horizontally. The mean accuracy for correctly identifying probe locations across experimental trials was 99% in the current sample. This is comparable with studies using the emotional face dot probe in controlled lab conditions (13). Researcher instruction in the lab, therefore, appears to be no more advantageous than on-screen instruction in terms of task accuracy, and an online version of the emotional face dot probe appears to be methodologically robust.

Informed consent was gained in all cases. All participants were entered into a prize draw to win an Apple iPad as compensation for their time. The study received all necessary approvals for it to take place in accordance with the Faculty of Health and Life Sciences Ethics Committee. Fully informed consent was provided by all participants.

### Treatment of data

#### Data reduction

Mean accuracy across experimental trials (i.e., correctly identifying probe location) was high at 99%, and this did not statistically differentiate groups (*t* = 0.31, df = 96, *p* = 0.76). Only reaction times for correct trials were taken forward for statistical analysis. Reaction times <200 ms, indicative of anticipatory responding, were eliminated, as were responses >1,000 ms, likely caused by lapses in concentration (47). Outlying responses on the dot-probe accounted for <1% of all experimental trials, and outlying responses did not differentiate groups (*t* = 0.33, df = 95, *p* = 0.74).

#### Attentional bias indices

Vigilance, characterized by speeding attention to negative stimuli, was calculated by subtracting mean response times for dots replacing neutral faces paired with another neutral face (i.e., neutral: neutral trials) from response times where dots replaced negative (sad) faces paired with neutral faces (i.e., valid sad: neutral trials). Positive scores, indicating faster speeding of attention to dots replacing sad faces compared with neutral faces when paired with another neutral face, indicated greater vigilance to negative stimuli. Disengagement, characterized by problems shifting attention away from negative stimuli, was calculated by subtracting mean reaction times for dots replacing neutral faces paired with sad faces (i.e., invalid sad: neutral trials) from response times where dots replaced neutral faces paired with another neutral face (i.e., neutral: neutral trials). Positive scores, indicating slower shifting of attention away from dots replacing sad faces compared with neutral faces when paired with another neutral face, indicated greater problems disengaging attention from negative stimuli.

### Statistical analysis

A series of bivariate and, for categorical variables, point bi-serial correlations were used to explore whether depression scores, and scores for attentional indices (vigilance and disengagement), might be related to sociodemographic and lifestyle variables, and characteristics of the autistic child. Group differences with respect to depression scores were assessed via one-way univariate ANOVA. MANOVA, supplemented with univariate tests, was used to explore whether groups might be differentiated with respect to vigilance and disengagement. Whether vigilance and disengagement might be related to depression scores was assessed with bivariate correlation.

## Results

### Control measures

Depression scores (all *p*s > 0.08), and scores for vigilance (all *p*s > 0.12) and disengagement (all *p*s > 0.11), were unrelated to almost all socio-demographic and lifestyle variables. Children living at home full time were the only exception; depression scores were higher for parents with more children living at home full time (*r* = 0.23, *p* = 0.02). Depression scores, and scores for vigilance and disengagement, were unrelated to the current age of the autistic child and age at diagnosis (all *p*s > 0.18). Number of children living at home full time, therefore, was controlled in relevant subsequent analyses.

### Depression scores and attentional bias

One way ANCOVA, adjusting for the number of children living at home full time, revealed depression scores [*F*_(1, 95)_ = 26.28, *p* < 0.001, *η_p_*^2^ = 0.22] were higher in caregivers. Caregivers’ mean depression score was 8.3 (SD = 2.6), placing them in the range for a borderline clinical case. Mean depression scores for controls, by contrast, were 5.1 (SD = 3.1), falling well within the normal range. Caregivers were in fact around 50% more likely, based on their depression scores, to meet the criteria for a borderline or clinical case (*χ*^2^ = 7.46, *p* < 0.01).

Wilks Lambda multivariate test for overall group differences in negative attentional bias was non-significant [*F*_(2, 95)_ = 0.67, *p* = 0.52, *η_p_*^2^ = 0.01]. Follow-up univariate tests revealed no group differences with respect to vigilance [*F*_(1, 96)_ = 0.64, *p* = 0.43, *η_p_*^2^ = 0.00] or disengagement [*F*_(1, 96)_ = 0.52, *p* = 0.47, *η_p_*^2^ = 0.00]. Vigilance and disengagement scores were also unrelated to depression scores (all *p*s > 0.50). Means and standard deviations for attentional bias indices and depression scores by group are displayed in Table 2.

**Table 2:** Means and standard deviations (M ± SD) for depression scores, and for vigilance and disengagement (milliseconds), by group.

	Caregivers (*N =* 57)	Controls (*N* = 41)	*p =*	*η_p_* ^2^
Depression scores	8.3 ± 2.6	5.1 ± 3.1	**<0.001**	0.22
**Attentional bias**				
Vigilance	0.70 ± 19.4	−2.19 ± 14.9	0.43	0.00
Disengagement	9.64 ± 0.29.9	5.85 ± 23.7	0.47	0.00

Vigilance = difference between neutral and congruent trials. Disengagement = difference between neutral and incongruent trials.

## Discussion

This study explored whether caring for an autistic child might be associated, in the form of vigilance or disengagement, with a negative attentional bias. Results revealed that caregivers’ depression scores were markedly higher compared with controls. This was in keeping with our original hypothesis and commensurate with other recent findings (1–4, 10). Caregivers’ mean depression scores were in fact >8.0, placing them in the range for a borderline clinical case. Mean depression scores for controls, by contrast, were 5.1, falling well inside the normal range. Caregivers were around twice as likely to satisfy the criteria for a borderline or clinical case based on their depression scores. The high depression scores characteristic of caring for an autistic child could have serious implications. Indeed, the relationship between caregivers’ emotional well-being and child autism severity has been shown to be bi-directional, with one affecting the other and vice versa. Autistic children of more depressed parents tend to do less well socially and behaviorally, forming fewer peer relationships and displaying more hyperactive and aggressive behaviors (47, 48). More depressed caregivers have also reported feeling less confident about providing good quality caregiving and being more likely to distance themselves from important caregiving tasks (49, 50), Finding ways to alleviate caregivers’ depressive symptomology, potentially improving the quality of life for the autistic child, should remain a high priority for future researchers.

With respect to attention, findings revealed caregivers were not more vigilant to, and no slower to disengage from, sad faces compared with controls. Caring for an autistic child, while associated with borderline clinical depression scores, does not seem to be characterized by an attentional bias for negative information. Moreover, vigilance and disengagement were unrelated to depression scores. These findings are incongruous with those from recent studies. Indeed, attentional errors, assessed via self-report and more objectively in the lab, were found to be greater in familial caregivers compared with controls (27–30). Several caregiving populations that match parents of autistic children for psychological stress and burden have also displayed attentional processing biases for negative information (32–35). Rumination, providing one psychological marker of, and closely related as a construct with, negative attentional bias, is also typically higher in the context of caring for an autistic child (36). Clinical depression is also one well-known psychological marker for negative attentional bias, and caregivers’ depression scores here averaged >8.0, satisfying a borderline clinical case.

Group disparities with respect to depression scores, but not attentional bias indices, might be explained by methodological choices. The dot-probe task here was configured for faces to appear for 500 ms only. Configuring the task so faces appeared for longer, for 1,000 ms, might have been more sensitive for detecting negative attentional biases. Indeed, whether attentional disparities can be observed between stressed and non-stressed samples has been shown to be moderated by stimuli presentation time. Caregivers of autistic children often report depression scores within the clinical range, and clinically depressed individuals were quicker to shift their attention to negative stimuli presented for 1,000 ms, but not 500 ms, compared with controls (51, 52). Whether attentional disparities between caregivers of autistic children and controls vary by stimuli presentation time, manifesting for stimuli presented for longer periods, could be investigated in future research. The negative attentional bias observed in depressed samples also tends to be a content-specific one. That is, it manifests for sad faces, as those congruent with the depressed person’s mood, but not for mood incongruent faces (e.g., anger). Whether the negative attentional bias characteristic of caring for an autistic child, to the degree one exists, might also be differentially affected by stimuli type, manifesting for certain types of faces and not others, could also be explored in the future. Infant faces, for example, preferentially engage the attentional systems of adults. For example, target detection on a visual search task was significantly slower when the target was paired with an infant, compared with an adult, face. The infant face, more than the adult face, interfered with task performance, slowing the detection of the visual target. This attentional sensitivity for infants’ faces, as evidenced by slower target detection, was also magnified for parents and when infant faces expressed negative emotions (53). The attentional system of adults, therefore, appears to be particularly sensitive to infants’ faces, and parents’ attentional system is particularly sensitive. The preferential attention of parents to infants’ faces has been explained as an evolutionary adaptation, ensuring that infants are attended to and, absent any developed language, receive essential caregiving (54). It has been estimated that language is absent or minimal in 25–35% of autistic children, with their intentions and needs instead communicated to parents instead via facial expressions and other non-verbal cues (46). Parents rely heavily on the nonverbal communication of their autistic child, especially facial expressions, and this seems to be reciprocal. Indeed, autistic children performed poorer than controls on emotion recognition tasks, finding decoding emotions from faces presented in photographs much more challenging (55). More recently, however, researchers demonstrated this effect was moderated by familiarity. That is, autistic children were poorer than controls at recognizing emotions on unfamiliar (i.e., stranger), but not familiar (i.e., parent), faces (56). Caring for an autistic child, therefore, might be associated with an attentional bias for faces displaying negative emotions, but this might be age-specific, observable for infant, but not adult, faces. Future research might explore this.

The findings reported here should be tempered by limitations. The dot-probe task had been intended to run in the lab, in controlled settings where researchers could deliver task instructions orally, but COVID-19 forced a change in study design. Accuracy rates for the online emotional face dot probe task used here were high (99%), matching those of lab-based protocols. Completion rates, by contrast, were poor, with 58.1% of participants failing to start the dot-probe task or complete it to a point where data could be used for analysis. Retention, however, was equally poor across the groups; caregivers were no more likely than controls to drop out at the dot probe stage. The high attrition rate resulted in a final sample of *N* = 98, and this failed to satisfy the conditions of the *apriori* power analysis. That said, a high rate of attrition was expected. Indeed, attrition rates are typically high for online studies, particularly where cognitively demanding tasks such as the one used here are incorporated (40, 41). Moreover, it is well known that caregivers of autistic children, making up more than half of the current sample, are a notoriously hard-to-reach population, with most of their time dedicated to the unremitting demands of caretaking. The findings reported here, therefore, should be interpreted cautiously until corroborated with a larger sample. Moreover, whether results from an online version of the emotional dot-probe task such as the one used here are robust, correlating with results from the same task completed in controlled lab settings, also remains to be seen, as does how the reliability of online protocols might vary as a function of the internet service provider. This might be a target for future research. Autism diagnosis in the current study was via parent report only, and a more formal diagnosis would have been better. Parent reports of autism diagnosis, however, do tend to be reliable (57). Much of the variation in caregivers’ depression scores has been found to be accounted for by characteristics of the autistic child, especially problematic behaviors and autism severity (58). These variables were not measured here, and this represents a notable limitation. Indeed, caregivers of children with greater behavioral problems typically report higher depression scores, and it might be this subset of caregivers who are particularly sensitive to negative attentional biases. All caregivers were recruited via online caregiving support groups, and the stress-alleviating effects of social support are well established (59, 60). The baseline level of social support in the current sample, therefore, might be higher than usual, and the current sample might not be representative. It might be that less socially supported caregivers, who also tend to report higher depression scores, are particularly sensitive to negative attentional biases. Finally, reaction time tasks such as dot probe quantify the time taken to shift attention to and from negative stimuli and, therefore, provide only indirect assessments of negative attentional bias. Eye tracking on the other hand captures the continuous flow of visual attention and, as a more rigorous protocol capturing a range of other attentional indices (e.g., fixation time, glance duration), might be more sensitive at detecting attentional biases.

In conclusion, caregivers of autistic children, while more depressed than controls, did not display an attentional bias for negative information. Attentional bias indices of vigilance and disengagement were also unrelated to depression scores. Whether attentional disparities between caregivers and controls might manifest with methodological adjustments, when negative stimuli are presented for longer periods and using more robust protocols involving eye tracking, might be explored in the future.

## Data availability statement

The raw data supporting the conclusions of this article will be made available by the authors, without undue reservation.

## Ethics statement

The studies involving human participants were reviewed and approved by Faculty of Health and Life Sciences Ethics Committee, Northumbria University. The patients/participants provided their written informed consent to participate in this study.

## Author contributions

BL designed the study, collected data, completed analysis, and write the paper. KM designed the dot probe task. PP collected data and helped with data analysis. MW helped design the study and co-wrote the paper. All authors contributed to the article and approved the submitted version.

## Conflict of interest

The authors declare that the research was conducted in the absence of any commercial or financial relationships that could be construed as a potential conflict of interest.

## Publisher’s note

All claims expressed in this article are solely those of the authors and do not necessarily represent those of their affiliated organizations, or those of the publisher, the editors and the reviewers. Any product that may be evaluated in this article, or claim that may be made by its manufacturer, is not guaranteed or endorsed by the publisher.
